# Recurrent Composite Lymphoma in an Elderly Man: A Case of Marginal Zone Lymphoma With Classic Hodgkin Features Treated With Brentuximab and Immunotherapy

**DOI:** 10.7759/cureus.84943

**Published:** 2025-05-28

**Authors:** Muhammad Daniyal, Anamm Polani, Amrat Kumar, Ahmad Raja, Marcy Canary

**Affiliations:** 1 Internal Medicine, Bassett Medical Center, Cooperstown, USA; 2 Hematology and Medical Oncology, Bassett Medical Center, Cooperstown, USA

**Keywords:** cancer immunotherapy, cd30+, epstein-barr virus-positive diffuse large b-cell lymphoma, follicular lymphoma, non-hodgkin's lymphoma

## Abstract

Composite lymphoma (CL) is a rare and diagnostically challenging condition where two or more distinct types of lymphoma coexist within the same anatomical site. We report the case of an 82-year-old man with a history of low-grade B-cell lymphoma, now presenting with recurrent disease characterized by a composite histology of marginal zone lymphoma (MZL) and classic Hodgkin's lymphoma (CHL). Management involved single-agent brentuximab due to comorbidities, followed by immune checkpoint inhibition upon relapse. This case illustrates key considerations in the diagnosis, staging, and treatment of CLs in geriatric patients. The clinical course further highlights the importance of personalized therapy selection based on age, comorbidities, and histological features. This case also contributes to the limited literature on real-world outcomes in patients with CL and underscores the need for future prospective studies and biomarker-driven treatment approaches in this rare entity.

## Introduction

Composite lymphoma (CL) is a rare hematologic condition characterized by the coexistence of two or more distinct lymphoma types within the same anatomical location or biopsy specimen. Although it comprises only 1%-4% of all lymphomas, its clinical importance lies in its diagnostic complexity and implications for treatment selection and prognosis [1]. The most frequently reported combination involves a B-cell non-Hodgkin's lymphoma (NHL), such as marginal zone lymphoma (MZL) or follicular lymphoma, paired with classic Hodgkin's lymphoma (CHL), as seen in this case [2].

The etiology and pathogenesis of CL remain incompletely understood, though several theories have been proposed. These include the possibility of biclonal or divergent clonal evolution from a common progenitor, genetic instability, prior immunosuppressive therapy, and viral involvement, particularly Epstein-Barr virus (EBV) in the Hodgkin component [3,4]. EBV is known to contribute to lymphomagenesis by promoting B-cell proliferation and survival, especially in immunosenescent or immunocompromised hosts, a relevant consideration in elderly patients [5].

From a diagnostic standpoint, CLs challenge conventional classification schemes. They often require extensive immunohistochemical analysis and molecular studies to distinguish and characterize each component. Immunophenotypic markers such as CD20, CD30, CD15, PAX5, Bcl-2, and CD3, in conjunction with EBV-encoded RNA (EBER) in situ hybridization, play a critical role in differentiating the B-cell and Hodgkin components [6]. Misdiagnosis or underdiagnosis can lead to suboptimal therapeutic strategies, particularly if only the indolent or aggressive component is identified.

Clinically, patients may present with B-symptoms, lymphadenopathy, or findings on surveillance imaging, as in our case. Management strategies for CL are not standardized due to its rarity and heterogeneity. However, treatment is typically guided by the more aggressive histologic subtype, which, in combinations involving CHLs, often necessitates CHL-directed therapy such as brentuximab vedotin or PD-1 inhibitors [7]. In elderly patients, particularly those with comorbidities or poor performance status, treatment must be adapted to minimize toxicity while maintaining disease control [8].

This case of an 82-year-old man with a past history of indolent low-grade B-cell lymphoma now presenting with a composite histology involving MZL and CHL underscores the importance of careful diagnostic evaluation and personalized treatment planning in geriatric oncology. This case also highlights the evolving role of targeted therapy and immunotherapy in managing complex lymphoid malignancies.

## Case presentation

An 82-year-old man with a history of stage IVA low-grade B-cell lymphoma, last treated in 2009 with rituximab and the cyclophosphamide, vincristine sulfate, and prednisone (CVP) regimen, presented for follow-up after experiencing a stroke in late 2021. A cervical spine CT scan incidentally revealed supraclavicular adenopathy. PET-CT confirmed lymphadenopathy in the cervical, thoracic, and retroperitoneal regions, along with presacral soft tissue thickening.

**Figure 1:**
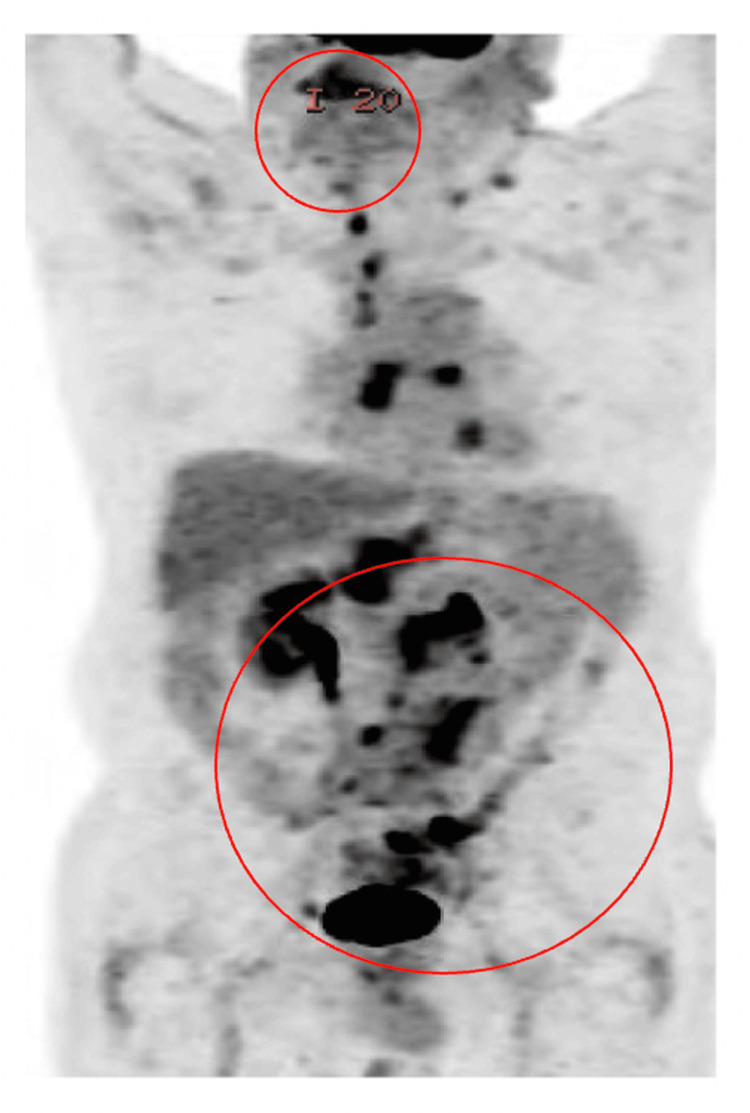
PET scan showing extensive fluorodeoxyglucose (FDG)-avid bilateral lymphadenopathy involving the lower cervical, mediastinal, retrocrural, and retroperitoneal regions (circles indicate hypermetabolic lymph nodes).

Given the patient’s chronic renal insufficiency and advanced age, treatment was initiated with a reduced dose of brentuximab vedotin. Rituximab was initially withheld due to previous intolerance. He showed excellent clinical response, with PET-CT scans taken in October 2022 and February 23 demonstrating no disease progression. A PET-CT taken in June 2023 (Figure 1) revealed a minor increased uptake in the sigmoid colon without clear nodal growth. Following 12 months of brentuximab, a repeat PET in early 2025 showed disease recurrence. Biopsy confirmed relapsed CL with persistent Hodgkin component.

**Figure 2:**
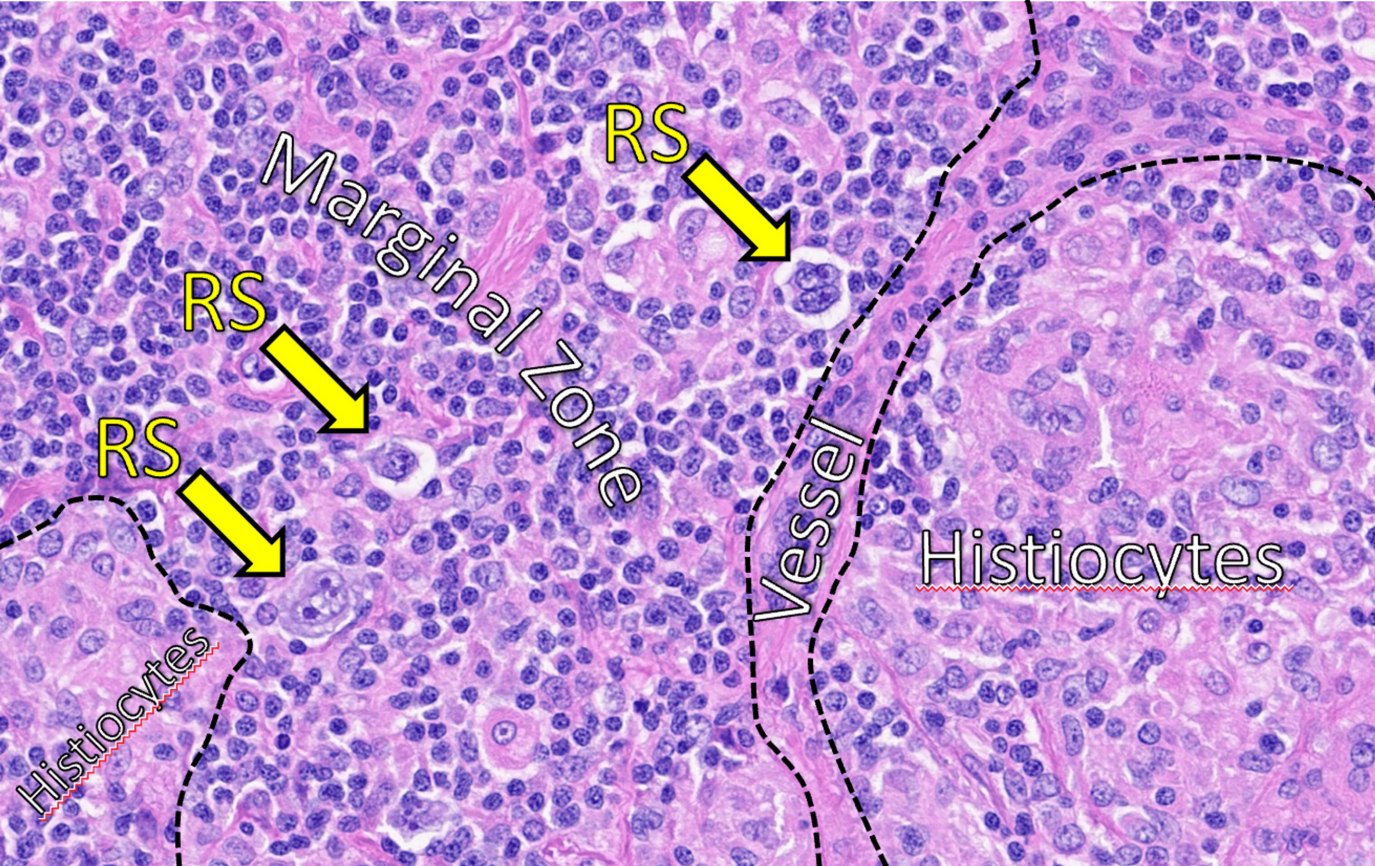
High-power H&E-stained section showing Reed-Sternberg (RS) cells within the marginal zone, adjacent to histiocyte-rich areas and a central vessel. Yellow arrows indicate RS cells. Findings are consistent with Epstein-Barr virus (EBV)-positive classic Hodgkin's lymphoma.

Excisional biopsy revealed CL composed of MZL (CD20+, CD5-, CD10-, Bcl-2+) with islands of EBV-positive Reed-Sternberg cells consistent with CHL (Figure 2). The disease was staged as IVB based on bilateral nodal and soft tissue involvement.

**Figure 3:**
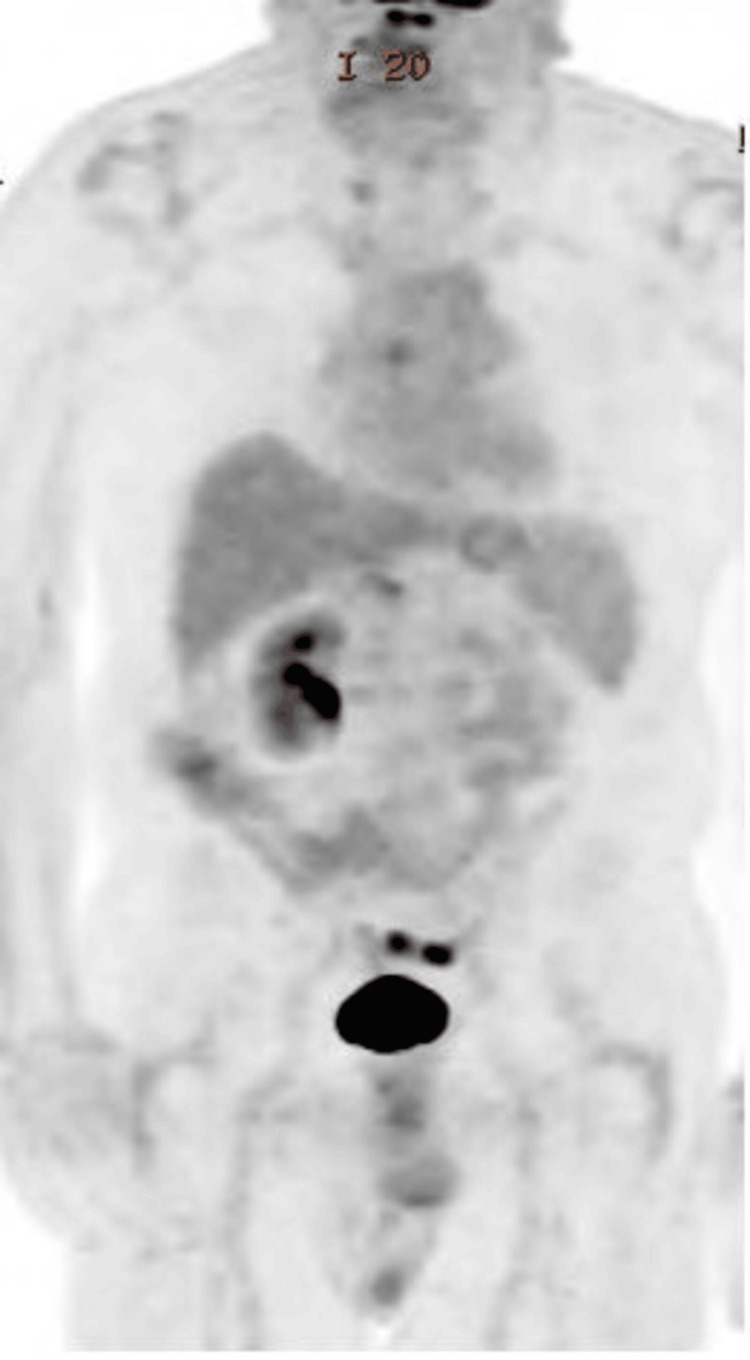
PET scan showing no evidence of FDG-avid disease. FDG: fluorodeoxyglucose.

Immune checkpoint inhibitor therapy with pembrolizumab was initiated. The patient was counseled on risks including fatigue, thyroid dysfunction, colitis, pneumonitis, and other immune-related adverse events. He consented to proceed with up to 24 months of immunotherapy. Currently, he remains under surveillance and is tolerating therapy. The follow-up PET scan showed no evidence of FDG-avid disease (Figure 3).

## Discussion

The diagnosis of CLs requires a comprehensive pathological workup including morphology, immunophenotyping, and EBV testing. In this case, classic marginal zone markers (CD20+, CD23+, CD43+, Bcl-2+) were seen alongside diagnostic Reed-Sternberg (RS) cells. EBV positivity further supported the diagnosis of CHL as part of the composite histology, consistent with previous findings suggesting a possible viral contribution to CHL transformation in CLs [4,7].

Therapeutic strategies for CL depend on the most aggressive histological component, typically the Hodgkin component, particularly in cases where CHL coexists with indolent NHL [5]. Brentuximab vedotin, an anti-CD30 antibody-drug conjugate, has shown substantial efficacy in relapsed CHL, especially in older or frail patients unable to tolerate ABVD chemotherapy [8]. The patient's good response to brentuximab supports its use as a frontline agent in CD30+ CLs with Hodgkin features.

Upon relapse, immune checkpoint inhibition was employed. Pembrolizumab, a PD-1 inhibitor, has demonstrated durable responses in relapsed/refractory CHL, with manageable toxicity profiles in elderly patients [9]. The decision to proceed with immunotherapy was influenced by the patient's stable organ function and relapse pattern, consistent with current clinical guidance [10].

Longitudinal PET-CT surveillance was essential in guiding treatment shifts, detecting minimal residual disease, and avoiding overtreatment, a key concern in geriatric oncology [11]. The role of PET-CT in composite lymphomas is evolving but remains critical in treatment response assessment and progression monitoring.

## Conclusions

This case illustrates the diagnostic complexity and therapeutic challenges of CL in elderly patients. Personalized treatment, beginning with single-agent brentuximab and later transitioning to immune checkpoint therapy, was critical in managing disease progression and maintaining quality of life. With no clear standard of care for CL, especially in the elderly, treatment must be individualized, balancing efficacy with tolerability.
